# Activation of antioxidant defences of human mammary epithelial cells under leptin depend on neoplastic state

**DOI:** 10.1186/s12885-018-5141-8

**Published:** 2018-12-18

**Authors:** Sinda Mahbouli, Jérémie Talvas, Audrey der Vartanian, Sophie Ortega, Stéphanie Rougé, Marie-Paule Vasson, Adrien Rossary

**Affiliations:** 1 Université Clermont Auvergne, INRA, UMR 1019, Unité de Nutrition Humaine, CRNH-Auvergne, F-63000 Clermont-Ferrand, France; 2 CHU Clermont-Ferrand, Centre Jean Perrin, Unité de Nutrition, CLARA, F-63000 Clermont-Ferrand, France

**Keywords:** Adipokines, Oxidative stress, Breast carcinogenesis, Cyclooxygenase, Glutathione, Heme-oxygenase, Lipid peroxidation

## Abstract

**Background:**

Obesity is associated with oxidative stress, a major factor in carcinogenesis, and with high leptin concentration. The aim of this study was to determine the effects of leptin on the antioxidant response in three human mammary epithelial cells each presenting a different neoplastic status: healthy human mammary epithelial cells (HMEC), oestrogen-receptor positive MCF-7 cells and triple-negative MDA-MB-231 cells.

**Methods:**

This in vitro kinetic study characterized the cell antioxidant response after 1, 6 and 24 h in the presence of leptin (10 or 100 ng/ml).The antioxidant response was defined in terms of cell glutathione content, gene expression and catalytic activity of antioxidant enzymes (i.e. glutathione peroxidase 1 (Gpx1), glutathione reductase (GR), glutathione S transferase (GST), heme-oxygenase 1 (HO-1) and cyclooxygenase-2 (COX-2)). Oxidative stress occurrence was assessed by lipid hydro peroxide (HPLIP) and isoprostane concentrations in culture media at 24 h.

**Results:**

At both concentrations used, leptin induced ROS production in all cell models, contributing to various antioxidant responses linked to neoplastic cell status. HMEC developed a highly inducible antioxidant response based on antioxidant enzyme activation and an increase in cell GSH content at 10 ng/ml of leptin. However, at 100 ng/ml of leptin, activation of antioxidant response was lower. Conversely, in tumour cells, MCF-7 and MDA-MB-231, leptin did not induce an efficient antioxidant response, at either concentration, resulting in an increase of lipid peroxidation products.

**Conclusions:**

Leptin can modulate the oxidative status of mammary epithelial cells differently according to their neoplastic state. These novel results shed light on oxidative status changes in mammary cells in the presence of leptin.

## Background

In obesity, accumulation of fat [1] is related to metabolic disorders [2], which are a risk factor for chronic diseases such as cancers [3]. Leptin, an adipokine upregulated during obesity, has been widely studied in carcinogenesis because of its many signalling pathways [4] involved in critical steps of pathogenesis such as cell proliferation [5, 6], inflammatory response [7] and modulation of the tumour environment [8]. Leptin is also known to reduce the efficacy of antioestrogen therapy [9]. Studies have clearly identified obesity, owing to the humoral secretions it entails, as a major risk factor in post-menopausal breast cancer [10]. However, very few studies have assessed the ability of these secretions to change cell metabolism with regard to oxidative status, especially that of primary healthy cells [11]. Oxidative stress is known to be involved in carcinogenesis [12], to modulate many cell signalling pathways [13] and to be linked to inflammation [14], but data are sparse on how leptin affects oxidative stress in breast cancer [15].

Because oxidative stress can be induced by obesity [16] and has a known role in carcinogenesis [12] we set out to study the oxidative status of different mammary epithelial cells. Our team’s previous work showed that leptin induced an inflammatory response in breast cancer in mice [17], and a different proliferative effect on neoplastic cells [5, 18]. We also showed that cytotoxicity of Natural Killer cells declined under leptin in obesity condition [19]. We hypothesized that between healthy and neoplastic cells, the different integration of the leptin signalling is due not only to their neoplastic status [20], but also to their oxidative status [21]. Regarding literature, plasma leptin concentrations were defined around 10 to 30 ng/ml and 50 to 150 ng/ml respectively for a lean and an obese adult woman [22]. Thus, we chose leptin doses at 10 ng/mL for physiological and 100 ng/mL for obese conditions, which are also relevant to tissue concentrations [8]. The aim of this work was thus to determine whether leptin at two concentrations would modulate oxidative status during a short 24-h time window, in terms of both oxidative production and antioxidant responses and subsequently would lead to an oxidative stress. Using healthy mammary epithelial cells (HMEC), and neoplastic MCF-7 and MDA-MB-231 cells, respectively known to be oestrogen-receptor-positive (ER+) and triple-negative metastatic cells, we characterized the cell antioxidant response. Among the antioxidant systems, we focused on the GSH metabolism, as it is the major cell antioxidant pathway. We investigated the mRNA expression and catalytic activity of the following antioxidant enzymes. Glutathione reductase (GR) reduces oxidized glutathione disulphide back to the reduced form GSH. Glutathione peroxidase 1 (GPx1) catalyses the reduction of harmful lipid peroxides in presence of GSH and protects the lipid membranes against oxidative damage [23]. Glutathione S Transferases (GSTs) are involved in cell detoxification by catalysing the conjugation of GSH to lipophilic compounds thereby increasing their solubility and excretion from the cell [24] and are involved in drug detoxifying by neoplastic cells [25]. Finally, heme oxygenase 1 (HO-1), a key regulator of cell redox homeostasis, becomes constitutive in neoplastic cells [26] and is strongly induced [26] to protect cells against toxic metabolites, oxidative stress and injuries [27–29]. In parallel, to assess the oxidative stress, intracellular protein thiol content and extracellular lipid peroxidation products such as lipid hydro peroxides (HPLIP) and isoprostanes (8-iso-PGF2α) were measured [30, 31]. Glutathione (GSH) and the redox state of protein thiols may overlap in the activation and regulation of many pathways such as kinases and transcription factors, besides compartmentalized functions [32, 33]. Lastly, the mRNA expression and catalytic activity of cyclooxygenase 2 (COX-2) were determined, as this enzyme is a well-known prognostic factor in early breast cancer [34] and makes a link between oxidative stress and inflammation [31]. Altogether, these markers characterize the antioxidant response to oxidative stress: high GSH content or antioxidant enzyme activities, induced by oxidative stress, define a protective state. Conversely, low GSH content or antioxidant enzyme activities, associated with a loss of inducibility to oxidative stress, define an aggressive state [24, 35].

## Materials and methods

### Cell culture

Healthy human mammary epithelial cells (HMEC, Caucasian woman aged 55 years, oestrogen receptor positive (ER+), progesterone receptor (PR) positive, human epidermal growth factor receptor 2 (HER2) positive; Lonza, Basel, Switzerland) grew in complete MEBM medium, supplemented with hydrocortisone (0.5 μg/ml), epithelial growth factor (10 ng/ml), insulin (5 μg/ml), gentamicin (50 μg/ml) / amphotericin-B (50 ng/ml) and bovine pituitary extract (0.4%) as recommended by the manufacturer (Lonza). Neoplastic human mammary epithelial cells were MCF-7 (ER+, PR+ and HER2+ cells from metastatic breast tumour of Caucasian woman aged 69 years), and MDA-MB-231 (ER-, PR-, HER2-, triple-negative cells from metastatic breast tumour of Caucasian woman aged 51 years). Neoplastic cells (ATCC, Molsheim, France) grew in RPMI 1640 medium (Biowest, Nuaillé, France) containing 10% foetal calf serum, _L_-glutamine (2 mM), penicillin (50 units/ml) and streptomycin (50 μg/ml) (Sigma-Aldrich, Saint-Quentin-Fallavier, France). Cultures were at 37 °C in a humidified atmosphere with 5% CO2.

### Treatment with leptin

Mammary epithelial cells (HMEC, MCF7 and MDA-MB-231), synchronized in serum free medium for 24 h before initiation of leptin treatment, grew for 0, 1, 6 or 24 h in their media either with or without recombinant human leptin (R&D, Abingdon, United Kingdom) at physiological (10 ng/ml) or obese (100 ng/ml) concentrations.

Cells were harvested after trypsinization and three phosphate buffer saline washes. Total cell lysates were obtained by two successive thawing-freezing cycles in TrisHCl 25 mM buffer pH 7.4 containing Tween 20 0.1% (Sigma-Aldrich, Saint-Quentin-Fallavier, France), with 15-s periods in an ultra-sound bath, and then stored at − 80 °C until analysis.

Proteins were quantified by the bicinchonic acid method (Interchim, Montluçon, France) using a standard curve with a bovine serum albumin solution (2 g/l), according to the manufacturer’s instructions.

### RNA isolation and reverse transcription

After treatment with leptin, total RNA were isolated from the epithelial cells by Trizol® reagent (Invitrogen, Saint-Aubin, France) according to the manufacturer’s protocol and quantified using a NanoDrop spectrophotometer (Nanodrop®2000, Thermo Scientific, Waltham, MA). Reverse transcription was performed in a thermocycler (Mastercycler ® gradient, Eppendorf, Montesson, France), on 1 μg of total RNA for each condition using a high-capacity cDNA reverse transcription kit (Applied Biosystems, Saint Aubin, France) with random hexamer pdN6 primers.

### Quantitative real-time PCR (qPCR)

qPCR was performed using SYBR®Green reagents according to the manufacturer’s instructions on a StepOne system (Applied Biosystems, Saint-Aubin, France). Each condition ran in triplicate. Relative quantification was obtained by the comparative CT method, based on the formula 2^-ΔΔCT^. Expression levels were normalized to the housekeeping gene (β actin) for each time point, and expressed as fold change from the basal expression level corresponding to untreated cells at time 0. Table 1 reports sequences and fragment sizes of the human-specific primers used for analysis.

**Table 1:** Summary of PCR primers

Gene name	Accession number	Primer sequences		Amplicon length
HO-1	BC_001491	5’ ACA-GTT-GCT-GTA-GGG-CTT-TA 3′	Foward	247 bp
		5’ CTC-TGA-AGT-TTA-GGC-CAT-TG 3′	Reverse	
GPx1	NP_002076	5’ GCA-CCC-TCT-CTT-CGC-CTT-C 3’	Foward	222 bp
		5’ TCA-GGC-TCG-ATG-TCA-ATG-GTC 3′	Reverse	
GR	BC_069244	5’ GTC-AGT-GGG-AAA-AAG-TAC-AC 3′	Foward	244 bp
		5′ GTA-CCT-TAT-CAT-GCC-GTA-TC 3′	Reverse	
COX-2	NM_000963	5’-TCT-CCT-TGA-AAG-GAC-TTA-TG −3’	Foward	198 bp
		5’-CAT-TGA-TGG-TGA-CTG-TTT-TA −3’	Reverse	
β actin	NM_001101	5’ TCG-TGC-GTG-ACA-TTA-AGG-AG 3′	Foward	262 bp
		5’ AGC-ACT-GTG-TTG-GCG-TAC-AG 3′	Reverse	

### Quantification of reactive oxygen species (ROS) production

After synchronization, human mammary epithelial cells, plated at a density of 22,500 cells/cm^2^ in a 96-well plate, incubated in the appropriate growth medium with dihydroethidine (2 μM) in the dark for 30 min at 37 °C. Experimentation started by the addition of leptin (10 ng/ml or 100 ng/ml) or medium (for control conditions). ROS production fluorescence was measured every 10 min over a 120-min window, using a microplate reader (Fluoroscan Ascent Microplate Fluorometer®, Thermo Scientific, Waltham, MA), set on an excitation wavelength of 485 nm and an emission wavelength of 520 nm. Fluorescence was corrected for background for each well.

## Enzyme activities

### Heme oxygenase

Heme oxygenase (HO-1) activity was measured by following the degradation of heme in biliverdin using the method of Shih and Yang modified [36]. Hemin (10 μM) transformation by cell lysate was determined in the presence of the buffer reagent (100 mM TrisHCl, 2 mM EDTA, 2 mM MgCl2, 0.5% Tween 20, 2 mM NADPH, pH 7.4) (Sigma-Aldrich, Saint-Quentin-Fallavier, France) by kinetic measurement at 405 nm and 37 °C, in a microplate spectrophotometer reader (Multiskan FC, Thermo Scientific, Waltham, MA). HO-1 activity was in IU/g of proteins using an extinction coefficient of 5.84 10^4^ cm^− 1^ M^− 1^ for hemin.

#### Glutathione reductase

Glutathione reductase (GR) activity was determined as described elsewhere [37]. The cell lysate was incubated with buffer reagent (100 mM Tris-HCl, 1 mM EDTA, 0.16 mM NADPH and 4.6 mM oxidized glutathione (GSSG), pH 7.4) (Sigma-Aldrich, Saint-Quentin-Fallavier, France). Kinetic NADPH oxidation was followed at 340 nm and 37 °C for 3 min in a microplate spectrophotometer reader. GR activity, normalized to the protein content, was in IU/g.

#### Glutathione peroxidase

Glutathione peroxidase (GPx) activity resulted in the oxidation of GSH in the presence of tert-butyl-hydroperoxide. Secondarily GR recycled GSSG in the presence of NADPH [38]. The cell lysate was incubated with buffer reagent (100 mM Tris-HCl, 1 mM EDTA, 22 mM tert-butyl-hydroperoxide, 5 mM GSH, 0.1 IU/ml GR, 2 mM NADPH, pH 7.4) (Sigma-Aldrich, Saint-Quentin-Fallavier, France). Kinetic NADPH oxidation due to GSH recycling was followed at 340 nm and 37 °C, in a microplate spectrophotometer reader. GPx activity, normalized to the protein content, was in IU/g.

#### Glutathione S-transferase

Glutathione S-transferase (GST) activity was quantified as previously described [37] using the conjugation reaction of GSH with artificial substrate 1-chloro-2,4-dinitrobenzene. The cell lysate incubated with buffer reagent (50 mM HEPES, 5 mM GSH, 1 mM 1-chloro-2,4-dinitrobenzene, pH 7.4) (Sigma-Aldrich, Saint-Quentin-Fallavier, France). The kinetic 1-chloro-2,4-dinitrobenzene-glutathionylation was followed at 340 nm and 37 °C, in a microplate spectrophotometer reader. GST activity, normalized to the protein content, was in IU/g.

#### Cyclo-oxygenase-2

Cyclo-oxygenase-2 (COX-2) activity was measured using a COX measurement kit from Cayman chemical (Cayman Chemical Company, Ann Arbor, MI). The 24 h time-point cell lysates incubated according to the manufacturer’s instructions. Kinetic measurement was performed at 590 nm and 37 °C, in a microplate spectrophotometer reader. COX-2 activity, normalized to the protein content, was in IU/g.

### Total glutathione

Total glutathione (GSH) content was determined by the method of Cereser et al. [39]. Briefly, dithiothreitol reduced cell lysate for 10 min at room temperature and glutathione ethyl ester was added as an internal standard. After protein precipitation, the supernatant was derivatized by adding ortho-phthal-aldehyde (OPA) (Sigma-Aldrich, Saint-Quentin-Fallavier, France). The HPLC separation of GSH–OPA adducts used a UP3 HDO C-18 reversed-phase silica column (150 × 3.60 mm, particle size 3 μm) from Phenomenex (Interchim, Montluçon, France) maintained at 37 °C followed by fluorimetric detection at 420 nm after excitation at 340 nm (Summit HPLC system, Dionex SA, Courtaboeuf, France). Derivatives eluted using a 10–50% acetonitrile gradient in a 25 mM phosphate buffer at pH 6 for 5 min. The flow rate was 0.25 ml/min for an elution run of 20 min. Chromatograms were integrated using Chromeleon software from Dionex (Version 6.80, Dionex SA, Courtaboeuf, France). GSH content, calculated using a standard curve plotted under the same conditions, was expressed in μmol/g of protein.

### Protein thiols

Protein thiols were assayed using the method described by Himmelfarb et al. [40]. Free thiol groups oxidized by dithiobis-2-nitrobenzoic acid (Sigma-Aldrich, Saint-Quentin-Fallavier, France) were measured at 405 nm on a microplate spectrophotometer reader. The cell lysate free thiol concentration was expressed as a ratio to protein content in μmol/g.

### Lipid peroxidation

#### Lipid hydro peroxides

Lipid hydro peroxide (HPLIP) quantification in 24 h time-point culture medium was obtained using the method described by Arab et al. [41]. Culture media were treated by buffer reagent (40 mM H_2_SO_4_, 20 mM formic acid, 150 μM iron _D_-gluconate and 120 μM xylenol orange in glycerol) (Sigma-Aldrich, Saint-Quentin-Fallavier, France). A standard curve was obtained using a tert-butyl-hydroperoxide solution. Measurements were made at 570 nm on a microplate spectrophotometer reader. The HPLIP concentration was in μmol/l.

#### Isoprostanes

Isoprostane (8-iso-PGF 2α) quantification in 24 h time-point culture medium was obtained using the STAT-8-isoprostane EIA measurement kit (Cayman Chemical Company, Ann Arbor, MI). Measurements were made according to the manufacturer’s instructions at 405 nm on a microplate spectrophotometer reader. The 8-iso-PGF 2α concentration was in ng/l.

### Statistical analysis

Each experiment was performed in triplicate and the average value treated as a single data point. Statistical analyses were performed using GraphPad Prism5 (GraphPad Software, Inc., La Jolla, CA). Data are expressed as means ± standard deviation. Between-group comparisons were performed by one- or two-way ANOVA as appropriate, followed by a Kruskal-Wallis or Bonferroni multiple comparison test. Level of significance was set at 0.05. Significances are indicated by different subscript letters or flagged as * *p* < 0.05, ** *p* < 0.01 and *** *p* < 0.001.

## Results

### Neoplastic breast cancer cells exhibit a lower antioxidative status compared to healthy mammary cells (Fig. 1)

Oxidative status is a major cell characteristic involved in the response to cell environment changes. The determination of cytosolic ROS production, of glutathione and protein-thiol contents and of antioxidant catalytic activities characterized the oxidative status of our different mammary epithelial cell models under basal condition (i.e. without leptin).

**Fig. 1:**
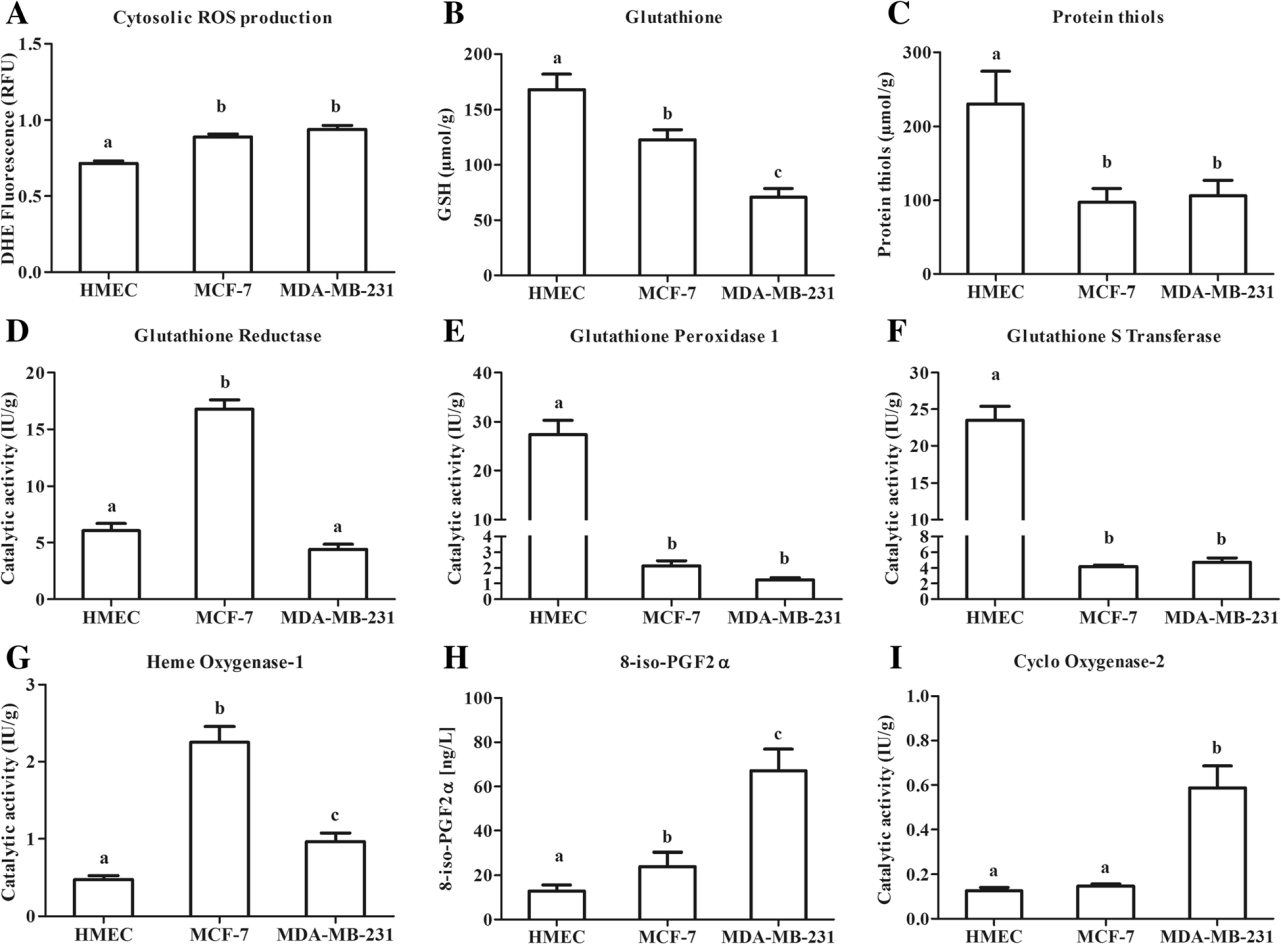
Basal oxidative status of human mammary epithelial cells. **a**: Basal fluorescence of dihydroethidine (DHE) for cytosolic ROS production. **b**: Basal cell content for total glutathione. **c**: Basal cell content for free protein thiols. **d**: Catalytic activity for glutathione reductase (GR). **e**: Catalytic activity for glutathione peroxidase 1 (GPx1). **f**: Catalytic activity for glutathione S-transferase (GST). **g**: Catalytic activity for heme oxygenase (HO-1). **h**: Basal production of 8-iso-PGF2α in culture medium. **i**: Catalytic activity for cyclooxygenase 2 (COX-2). Values are expressed as mean ± standard deviation (*n* = 6). Between-groups comparison was performed by one-way ANOVA followed by the Kruskal-Wallis multiple comparison test. The significance level was set at 0.05. Different letters (a ≠ b ≠ c, *p* < 0.05) indicate statistical significance between groups

As usually reported, cytosolic ROS production of superoxide anion (O_2_^°-^) was higher in neoplastic cells than in healthy primary cells (*p* < 0.05, Fig. 1a). Healthy cells also had a higher glutathione content (168 ± 31 μmol/g) than neoplastic cells (− 73% in MCF-7 and - 42% in MDA-MB-231 compared with HMEC, *p* < 0.05, Fig. 1b). A similar pattern was observed for the protein-thiol content (*p* < 0.05, Fig. 1c). This results in a higher redox potential in healthy cells than in neoplastic cells.

GR catalytic activity was 3-fold higher in MCF-7 than in HMEC and MDA-MB-231 (Fig. 1d). HMEC had a 10-fold higher GPx1 catalytic activity, and a 5-fold higher GST catalytic activity than the neoplastic cells (p < 0.05, Fig. 1e and f). Conversely, neoplastic cells significantly induced HO-1 catalytic activity (4-fold higher in MCF-7 and 2-fold higher in MDA-MB-231 than in HMEC, *p* < 0.05, Fig. 1g).

The imbalance between pro- and anti-oxidative systems resulted under basal conditions in an increase in 8-iso-PGF 2α content in neoplastic cell media (*p* < 0.05, Fig. 1h), highlighting a constitutive oxidative stress in these cells even in the absence of leptin. In addition, MDA-MB-231 cells presented a higher COX-2 catalytic activity than the other cells (Fig. 1i).

Our models presented three different levels of oxidative status. A protective redox defence in the healthy cells characterized by high defence levels. Conversely, triple negative MDA-MB-231 cells showed an aggressive pro-oxidant and inflammatory state. The neoplastic ER+ MCF-7 cells presented an intermediary profile.

### Leptin induces an antioxidant response in healthy human mammary epithelial cells but not in the neoplastic cell lines (Figs. 2 and 3)

As healthy and neoplastic cells presented a different basal oxidative status, these cells could integrate the leptin signal differently. We therefore focused on the modulation of oxidative status with 10 and 100 ng/ml of leptin. The two aspects of the oxidative response studied were the non-enzymatic and the enzymatic antioxidant response.

**Fig. 2:**
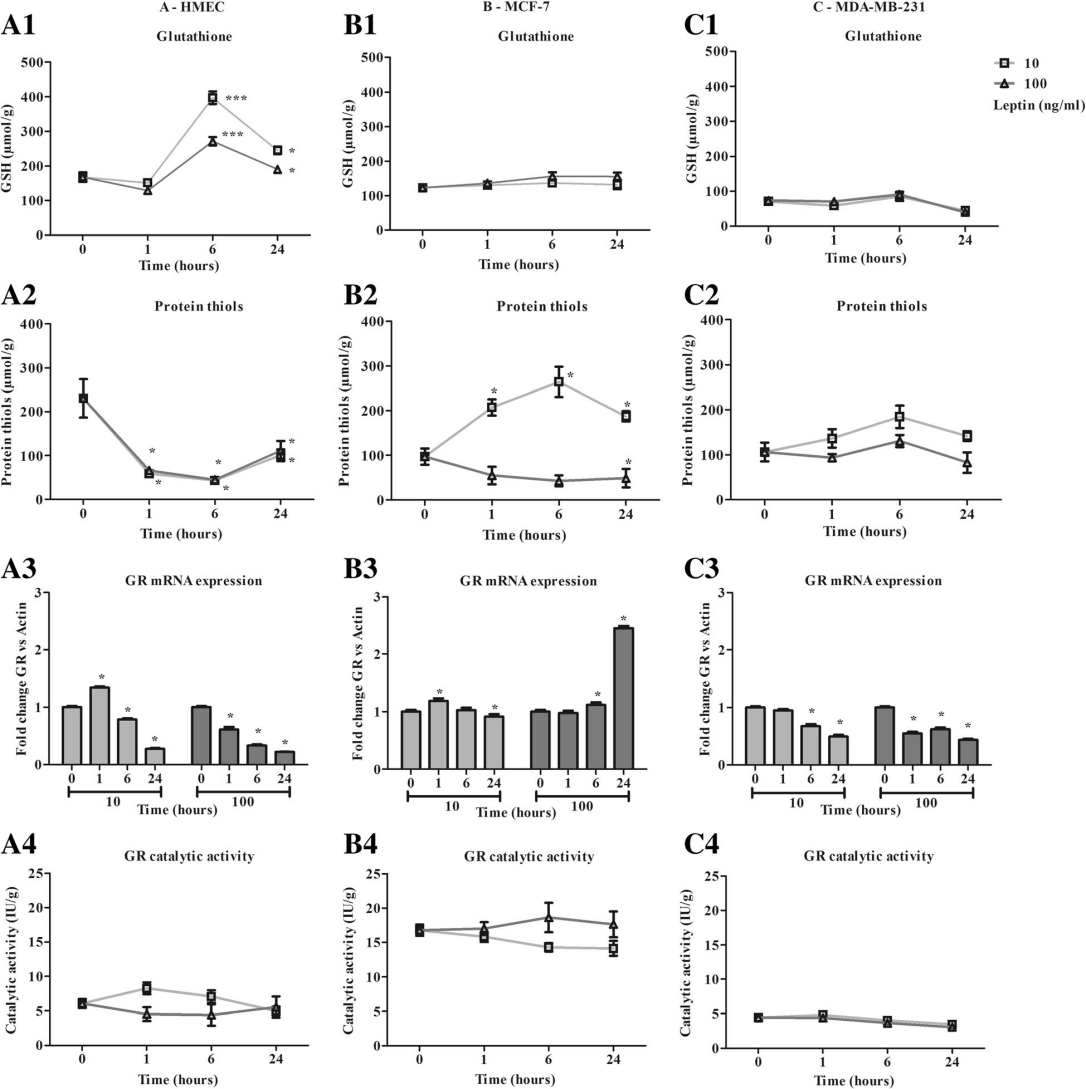
Characterization of antioxidant response time course in the presence of leptin (10, 100 ng/ml) in human mammary epithelial cells. **a**: Antioxidant response of HMEC. **b**: Antioxidant response of MCF-7. **c**: Antioxidant response of MDA-MB-231. In terms of: 1- Total glutathione, 2- Protein thiols, 3- Glutathione reductase mRNA expression 4- Glutathione reductase catalytic activity. Values are expressed as mean ± standard deviation (*n* = 6). Between-groups comparison was performed by one-way ANOVA followed by the Kruskal-Wallis multiple comparison test. The significance level was set at 0.05. Statistical significance between groups is indicated by * *p* < 0.05, ** *p* < 0.01 and *** *p* < 0.001

**Fig. 3:**
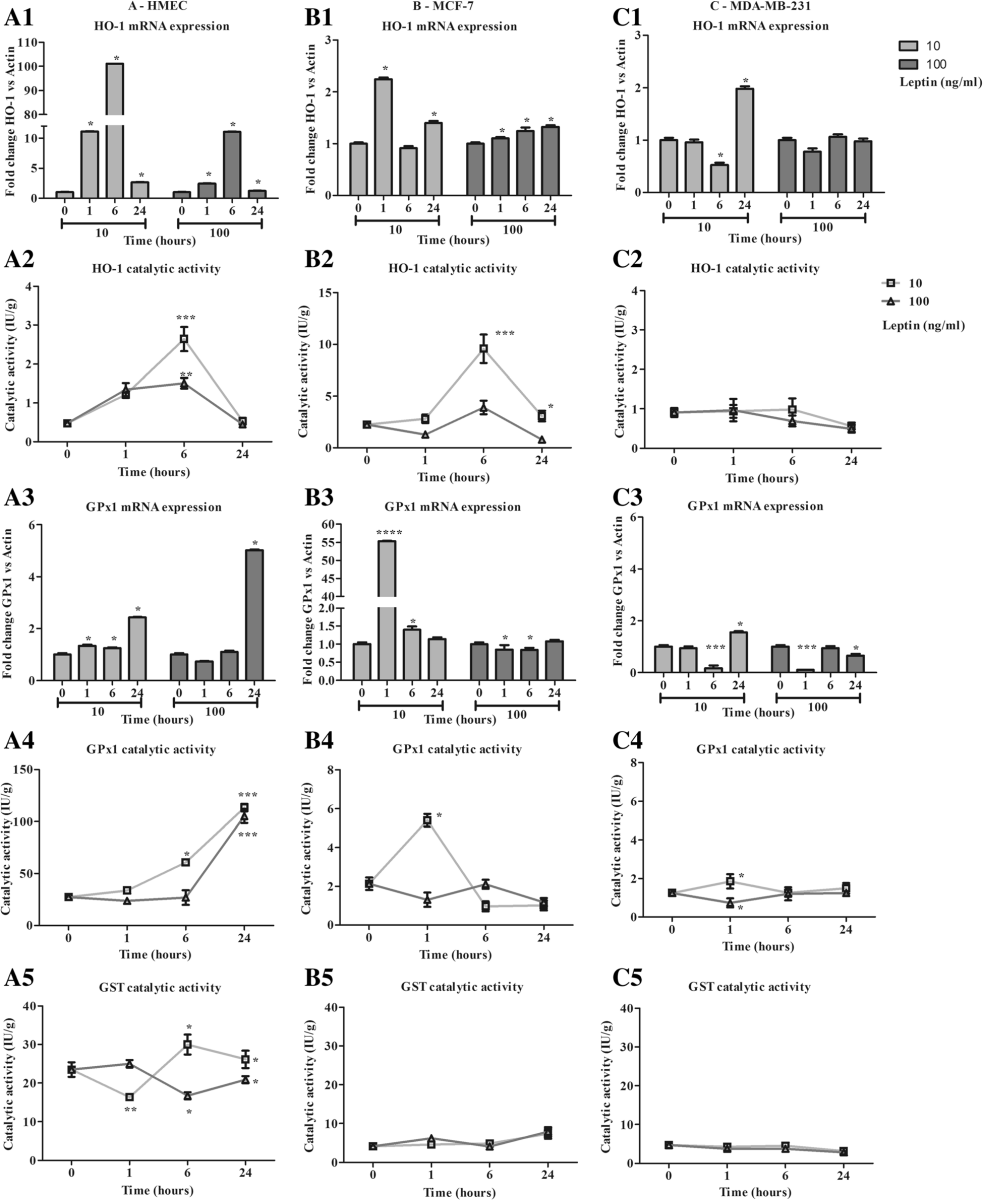
Characterization of antioxidant response time course in the presence of leptin (10, 100 ng/ml) in human mammary epithelial cells. **a**: Antioxidant response of HMEC. **b**: Antioxidant response of MCF-7. **c**: Antioxidant response of MDA-MB-231. In terms of: 1- Heme oxygenase 1 mRNA expression, 2- Heme oxygenase 1 catalytic activity, 3- Glutathione peroxidase 1 mRNA expression, 4- Glutathione peroxidase 1 catalytic activity, 5- Glutathione S-transferase catalytic activity. Values are expressed as mean ± standard deviation (*n* = 6). Between-groups comparison was performed by one-way ANOVA followed by the Kruskal-Wallis multiple comparison test. The significance level was set at 0.05. Statistical significance between groups is indicated by * *p* < 0.05, ** *p* < 0.01 and *** *p* < 0.001

### Non enzymatic antioxidant response

Cell GSH content increased in HMEC at 6 h from 168 ± 31 μmol/g to 398 ± 40 μmol/g and 272 ± 27 μmol/g with 10 and 100 ng/ml of leptin respectively. GSH content then decreased at 24 h (Fig. 2 A1). Protein thiols content decreased from 231 ± 116 μmol/g to around 44 μmol/g at 6 h, and then increased up to 100 μmol/g at 24 h for both concentrations of leptin (Fig. 2 A2). At both concentrations, leptin induced a change in the redox potential of healthy cells.

GSH content remained stable in MCF-7 and MDA-MB-231 with 123 ± 21 μmol/g and 71 ± 20 μmol/g respectively (Figs. 2 B1 and C1) at both leptin concentrations used. For 10 ng/ml of leptin, protein thiols content increased from 97 ± 69 μmol/g to 187 ± 17 μmol/g at 24 h, whereas a slight decrease was observed with 100 ng/ml of leptin (Fig. 2 B2) in MCF-7. In MDA-MB-231, no significant effect on protein thiol content was observed (Fig. 2 C2). Taken together, leptin seemed to have a weak effect on redox potential in any of the neoplastic cell models.

### Enzymatic antioxidant response

At both leptin concentrations, the expression of GR decreased steadily from 1 h to 24 h with no modification of the catalytic activity, which remained stable in HMEC (Fig. 2 A3). In MCF-7, GR expression remained quite stable until 24 h except for a 2.45-fold increase with 100 ng/ml of leptin (Fig. 2 B3, *p* < 0.05). However, GR catalytic activity remained stable during the time course and was 3-fold greater than in the other cell models (Fig. 2 B4). In MDA-MB-231, GR expression slightly decreased, and its lowest catalytic activity remained stable (Fig. 2 C3 and C4).

The time course of HO-1 expression in HMEC showed a very marked increase at 1 h and 6 h followed by a decrease at 24 h characterized by a higher level for 10 ng/ml than for 100 ng/ml of leptin (*p* < 0.05, Fig. 3 A1). As expected, catalytic activity increased from 1 h to 6 h (*p* < 0.05, Fig. 3 A2). In neoplastic cells, mRNA expression increased at a low level (MCF-7: 2.2 ± 0.1-fold expression at 1 h; MDA-MB-231: 2.0 ± 0.1-fold expression at 24 h, Figs. 3 B1 and C1), resulting in MCF7 in an increase in catalytic activity at 6 h to 9.6 ± 3.1 IU/g for 10 ng/ml of leptin (Fig. 3 B2). In MDA-MB-231, activity remained stable at 1.0 ± 0.4 IU/g from 0 to 6 h then slightly decreased at 24 h (Fig. 3 C2), underscoring the loss of inducibility of this enzyme in the neoplastic cells.

In HMEC, leptin induced an increase in GPx1 expression at 24 h to 2.4 ± 0.1- and 5.0 ± 0.1-fold expression (Fig. 3 A3). That resulted in marked activation of the catalytic activity from 27.3 ± 2.9 IU/g to around 110 IU/g at 24 h (p < 0.05, Fig. 3 A4). In neoplastic cells, mRNA expression was induced principally for MCF-7 at 1 h with 10 ng/ml of leptin, which resulted in a time-limited increase in catalytic activity (Fig. 3 B4). In MDA-MB-231, a very marked but time-limited decrease in expression was observed at 6 h at both leptin concentrations, with no effect on catalytic activity (Fig. 3 C4).

A slight increase to 26.1 ± 2.3 IU/g and a slight decrease to 20.9 ± 0.9 IU/g were observed in the GST catalytic activity of HMEC at 24 h for 10 ng/ml and 100 ng/ml of leptin, respectively (Fig. 3 A5), whereas no significant change was observed in neoplastic cells (Fig. 3 B5 and C5).

Leptin induced antioxidant defences in healthy cells, with less efficacy under the greater concentration. Whereas, leptin resulted in a partial induction of antioxidant defences in neoplastic ER+ cells only at the lower concentration and had no effect on the triple-negative cells.

### Leptin results in an inflammatory signalling in the three cell lines and an oxidative stress via lipid peroxidation in neoplastic cells only (Fig. 4)

Imbalance between pro- and anti-oxidative responses could result in lipid peroxidation related to a pro-inflammatory status favouring carcinogenesis and tumour growth. Lipid peroxidation products in culture medium and cell COX-2 activity were therefore assayed in our models.

**Fig. 4:**
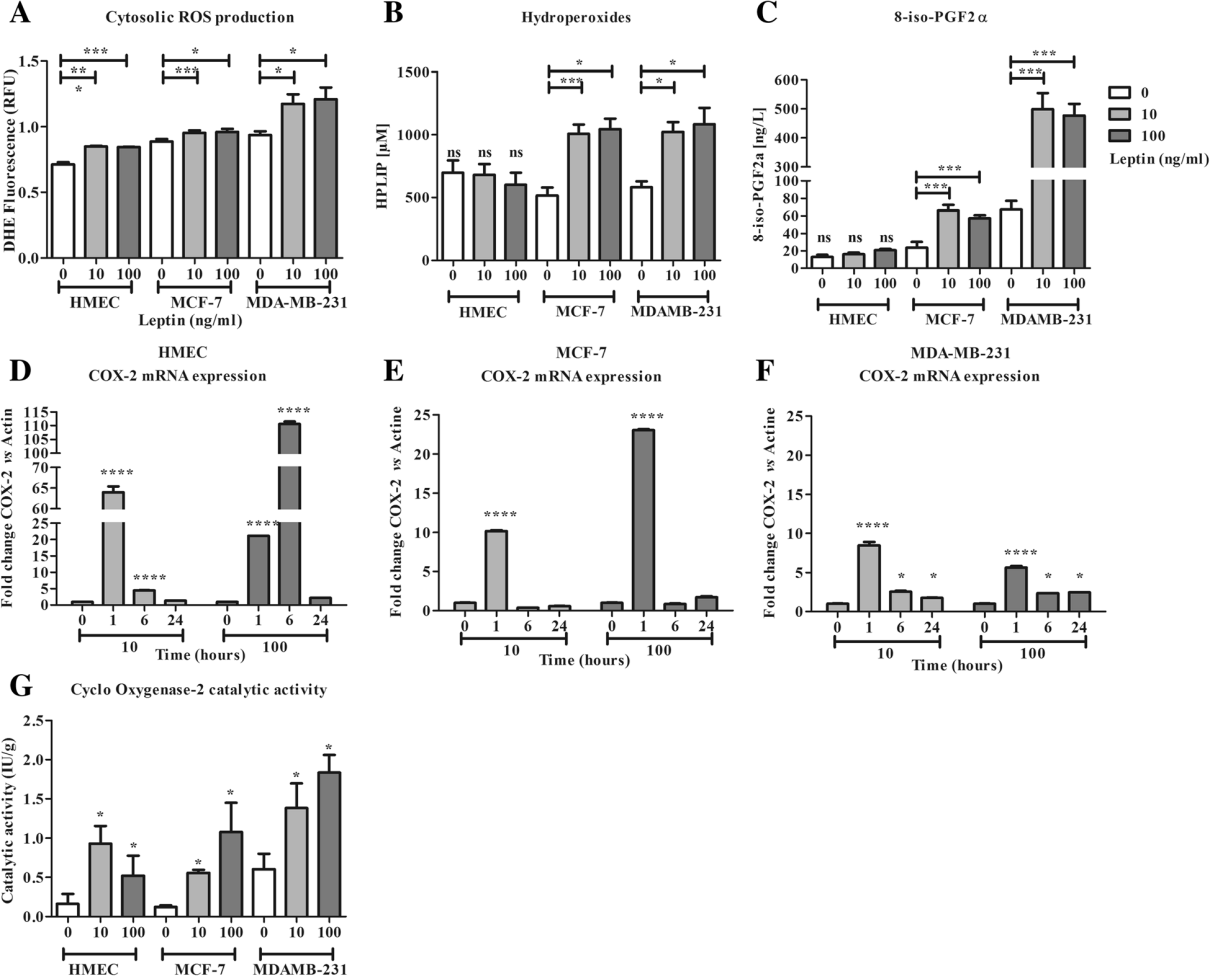
Characterization of non-enzymatic and enzymatic lipid peroxidation in the presence of leptin. **a**: Cell production at 10 min for cytosolic ROS production (DHE). **b**: Evaluation of lipid hydroperoxides in culture medium at 24 h. **c**: Evaluation of 8-iso-PGF2α in culture medium at 24 h. **d**: mRNA expression of cyclo-oxygenase 2 (COX-2) in HMEC. **e**: mRNA expression COX-2 in MCF-7. **f**: mRNA expression of COX-2 in MDA-MB-231. **g**: Catalytic activity of COX-2 in cells at 24 h. Values are expressed as mean ± standard deviation (n = 6). Between-groups comparison was performed by one-way ANOVA followed by the Kruskal-Wallis multiple comparison test. The significance level was set at 0.05. Statistical significance between groups is indicated by * *p* < 0.05, ** *p* < 0.01, *** *p* < 0.001 and **** *p* < 0.0001

In all cell lines, cytosolic ROS production increased in the presence of leptin (119, 107 and 125% of basal production, respectively for HMEC, MCF-7, MDA-MB-231; Fig. 4 a). In response to the ROS production and the antioxidant response (from the activation of GPx1), at 24 h and at either dose of leptin, in HMEC, no lipid peroxidation was observed, HPLIP and 8-iso PGF2α contents remaining stable in the culture medium (Fig. 4 b and c). An overexpression of COX-2 mRNA at 1 h and 6 h was observed and resulted in a significant increase in COX-2 activity at 24 h (Fig. 4 d and g).

Conversely, in neoplastic cells, a 2-fold increase in HPLIP content was observed (Fig. 4 b). In the same way, 8-iso-PGF2α content increased for both concentrations of leptin in the two neoplastic cell lines at a higher level for MDA-MD-231 cells (Fig. 4 c). At both concentrations of leptin, an overexpression of COX-2 mRNA at 1 h was observed in all cell lines (Fig. 4 d, e and f). In MDA-MB-231 the overexpression observed at 1 h persisted up to 24 h (Fig. 4 f). These mRNA expression increases resulted in an increase in COX-2 activity at 24 h, which was greater at the concentration of 100 ng/ml of leptin in the neoplastic cells (Fig. 4 g).

Finally, leptin signalling resulted in a pro-inflammatory environment associated to lipid peroxidation in neoplastic cells, whereas antioxidant defence induction prevented lipid peroxidation of healthy cells.

## Discussion

Several studies have investigated the impact of leptin in carcinogenesis in terms of cell proliferation [5], invasiveness [6], or angiogenesis and cell metabolism [42], but few have focused on the oxidative status of mammary neoplastic cells [43] despite some evidences in healthy cells [44]. In the present study, we compared the effect of leptin on oxidative status in three cell lines representing different neoplastic states, from healthy to metastatic.

On the basal condition, our models presented three different levels of oxidative status, from a protective redox defence in the healthy cells (characterized by higher cell concentrations of glutathione and enhanced GPx and GST catalytic activities) to an aggressive pro-oxidant and inflammatory state in MDA-MB-231 (lower antioxidant defences and higher COX-2 activity) [45, 46]. The neoplastic MCF-7 cells presented an intermediary profile characterized by high catalytic activities for GR and HO-1 but lower GSH and free protein thiol contents. We also showed that cytosolic ROS production at basal level was higher in neoplastic cells than in healthy mammary cells. Many of our observations are in line with biological characteristics of these cell lines [46, 47]. Mitochondrial ROS production was greater with a very low cytosolic ROS production, consistent with cell metastatic ability [48]. HO-1 lost its induction capacities, and its basal catalytic activity was greater than that of the healthy cells [26]. Nonetheless, this inducible enzyme became constitutive in neoplastic cells owing to a permanent transcription [29, 49] as shown in MCF-7 and MDA-MB-231 cells. The COX-2 expression and catalytic activity was greater in MDA-MB-231 cells than in the other cells [45]. Also, the stress resulting from a greater ROS production and a lower antioxidant defence protection [50], leads to an increase in the pro inflammatory product 8-iso-PGF2α. These results are in line with the constitutive oxidative stress widely observed in cancer cells [10, 14] which promotes tumorigenesis, vascularization and cell growth [51].

Many of the leptin signalling pathways, such as STAT3, PI3K-AKT or MAPK, are redox-sensitive and can be modulated by the oxidative status [8]. Oxidative status is a powerful mechanism for the regulation of various aspects of cell metabolism and functions such as stress defences [13], oestrogen receptor activity [52], and cell proliferation or inflammation [53]. GSH and protein thiols may have overlapped effects in the regulation of many signalling pathways through redox state of kinases and transcription factors [32, 33]. In the field of leptin signalling, p53 [54] and STAT3 [21] are regulated by their disulphide oxidative status and glutathionylation [32]. In addition, the PI3K-AKT pathway, one of the canonical leptin pathways [55], can stimulate NF-κb involved in the antioxidant response and cell growth [50, 56]. In this light, the differences in basal oxidative status between healthy and neoplastic cells could contribute, via the disulphide oxidative status of the signalling pathway, to different responses to leptin [57].

As leptin is able to increase cell ROS production in our cell lines, the antioxidant response was characterized in our cell lines after leptin stimulation.

**Fig. 5:**
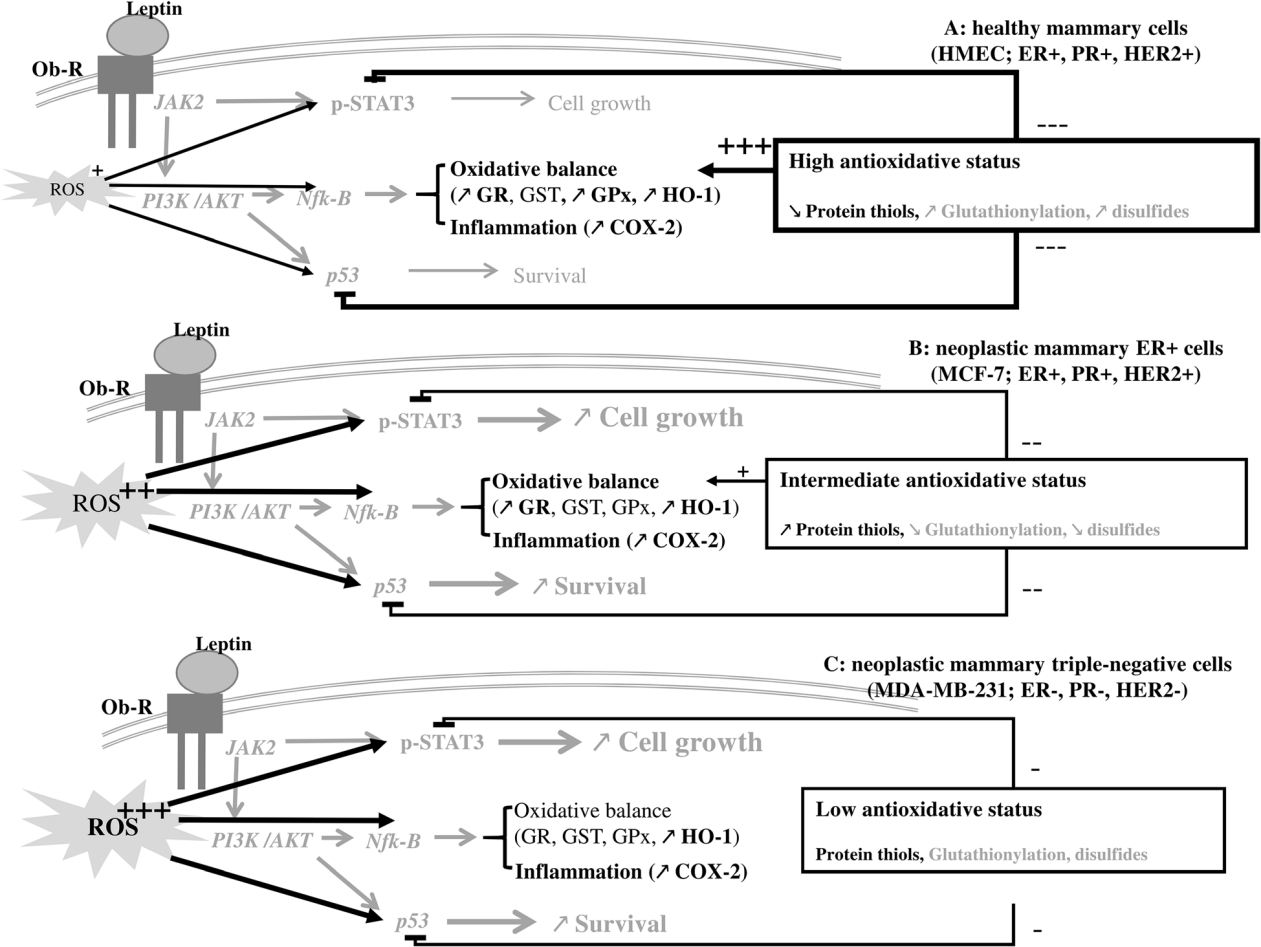
Modulation of oxidative status and leptin signalling pathway in human mammary epithelial cells. Leptin, via its receptor Ob-R, activated several signalling pathways such as PI3K/AKT parallel to JAK/STAT cascade. This resulting in modulation of cell growth, cell survival, oxidative status and inflammation. Depending of the neoplastic status of cells, oxidative status could also modulate these pathways through glutathionylation and protein disulphides. **a**: in healthy cells, due to the high antioxidant status, growth and survival pathways could be attenuated by glutathionylation and protein disulphides. Thus, leptin signalling favours preferentially the induction of antioxidant defences and of inflammation. **b**: in neoplastic ER+ cells, the intermediate antioxidant status was not sufficient to attenuate cell growth and survival pathways. Thus, in the presence of leptin all the signalling pathways could be induced, resulting in a stimulation of growth, survival, antioxidant defences and inflammation. **c**: in triple-negative cells, the high ROS production associated to the lower antioxidant status could favour growth, survival, and inflammation pathways in presence of leptin. Results of this study are draw in black. Data from previous report are shown in grey. Stimulation effect is flagged by + and inhibitor effect is flagged by -. JAK: Janus kinase; Ob-R: leptin receptor; PI3K: phosphor-inositol-3-kinase; STAT3: signal transducer and activator of transcription 3; GR: glutathione reductase; GST: glutathione S transferase; GPx: glutathione peroxidase; HO-1: heme oxygenase 1; COX-2: cyclo-oxygenase 2

At both leptin concentrations, HMEC exhibited a strong antioxidant response due to the mRNA overexpression of the major antioxidant enzymes (HO-1, GPx1), which results in an increase in their catalytic activity. Owing to the activation of GPx1, no increase was observed in the pro-inflammatory products of lipid peroxidation such as HPLIP or the non-enzymatic 8-iso-PGF2α. However, in presence of leptin, a stimulation of both mRNA expression and catalytic activity of COX-2 appeared and conducted to an inflammatory response. In addition, a considerable increase in GSH was observed and the protein thiols were oxidized, which are evidence of an antioxidant defence [57, 58] and of cell adaptation to the environment [59]. However, in presence of 100 ng/ml of leptin, the antioxidant response was lower in healthy cells. Indeed, there was a lower production of GSH, along with a lack of activation of GST and GR activities and a lower activation of HO-1 and GPx1. The activation of COX2 remained significant. Thus, leptin, known to potentiate cell growth in mammary neoplastic cells [8], seems to be integrated as a threat signal in healthy cells, resulting in the enhancement of the antioxidant defences associated to an inflammatory response. In light of these results, we could hypothesize that in healthy cells, high antioxidative status could favour the NF-κb signalling to enhance antioxidant and inflammatory response, and could inhibit the STAT3 and p53 signalling through glutathionylation. That could explain the huge decrease in protein thiols (Fig. 5a). Moreover, in obese condition, hyperleptinemia favours a pro-inflammatory environment and reduces the antioxidant defence of healthy cells [60]. Thus the healthy tissue could be less efficient in counteracting neoplastic cell growth and anti-tumour signalling [6].

In our conditions, as in previous observations [49, 57, 58, 61, 62], MCF-7 exhibited an incomplete antioxidant response differently modulated by leptin. At the lowest concentration of 10 ng/ml of leptin, the efficacy of the antioxidant response was related to the induction of HO-1 and GPx1, and the high GR activity (3 times higher than that of the other cells) and to the availability of protein thiols, as shown by the cell content increase. That can reflect a less glutathionylation and/or disulphide level in proteins. However, at 100 ng/ml of leptin, none of these changes was observed except a late GR mRNA expression increase. This observation could be a transcription regulation to maintain the GR mRNA pool and later the high level of activity, specific in this cell line. Despite an induction of lipid peroxidation defence, leptin signalling resulted in a substantial increase in oxidative damage at both concentrations shown by the majored lipid peroxidation. Moreover, the overexpression of COX-2 was associated with a greater increase in its catalytic activity at both leptin concentrations. This enhanced COX-2 activity and the 8-iso-PGF2α production reflect an inflammatory response to leptin favouring neoplastic cell growth [30, 31]. In the case of MCF-7, the intermediate antioxidative status could result in a less activation of NF-κb signalling, lighted by the lower antioxidant response, and could activate STAT3 and p53 signalling as suggested by the increased protein thiol content (Fig. 5b). Thus our results confirm the implication of leptin signalling in cell growth as previously described [5], and in pro-inflammatory response in ER+ neoplastic cells (Fig. 5b). These data confirm the poorer prognosis observed in breast cancer due to obesity [7], and highlight the role of leptin in this matter [8, 62].

Finally, whatever the concentration used, leptin did not affect the antioxidant response of the triple-negative MDA-MB-231 cells. At both concentrations, these cells still have an aggressive phenotype with low antioxidative status and constitutive activities of COX-2 and HO-1. Moreover, leptin signalling resulted in no change of glutathione or protein thiol contents. This low antioxidative status associated with majored ROS production could favour inflammatory, growth and survival pathways (Fig. 5c), as previously described by our team [5]. All these observations were consistent with the most aggressive profile of this cell line [62, 63] which is reinforced in obesity as shown in clinical observations [47].

## Conclusions

This in vitro study helps to gain a better understanding of the oxidative defence in human mammary epithelial cells in response to leptin signalling depending on cell-neoplastic status. Healthy cells exhibit an inducible antioxidant response, which is less effective under the hyperleptinemia condition. Conversely, in neoplastic cells, whether ER+ or triple-negative, leptin signalling results in a lack of antioxidant response related to a major oxidative stress and a pro-inflammatory response. These observations support our previous results and indicate that leptin could affect antioxidant status of healthy cells and favour a permissive environment for neoplastic cells. Our novel findings point to a potential link between obesity-related leptin secretion and breast cancer through cell oxidative status, which contributes to the aggravating effect of obesity in breast carcinogenesis. Taken together these data shed new light on the impact of leptin in carcinogenesis and support the utility of modulating leptin signalling pathways and oxidative status in obese patient to obtain health benefits.

## Abbreviations

8-iso-PGF2α: isoprostane
COX-2: Cyclo-oxygenase-2
DHE: Dihydroethidine
ER+: Oestrogen receptor positive
GPx1: Glutathione peroxidase 1
GR: Glutathione reductase
GSH: Glutathione
GST: Glutathione S-transferase
HER2: Human epidermal growth factor receptor 2
HMEC: Human mammary epithelial cells
HO-1: Heme oxygenase-1
HPLIP: Lipid hydroperoxides
Ob-R: Leptin receptor
OPA: Ortho-phthal-aldehyde
PR: Progesterone receptor
qPCR: Quantitative real-time PCR
RFU: Relative fluorescence unit
ROS: Reactive oxygen species

## References

[1] Ceddia RB (2005). Direct metabolic regulation in skeletal muscle and fat tissue by leptin: implications for glucose and fatty acids homeostasis. Int J Obes.

[2] Gregor MF, Hotamisligil GS (2007). Thematic review series: adipocyte biology. Adipocyte stress: the endoplasmic reticulum and metabolic disease. J Lipid Res.

[3] Vargas-Hernández VM, Vargas-Aguilar V, Moreno-Eutimio MA, Acosta-Altamirano G, Tovar-Rodriguez J (2013). Metabolic syndrome in breast cancer. Gland Surg.

[4] Guo S, Liu M, Wang G, Torroella-Kouri M, Gonzalez-Perez RR (2012). Oncogenic role and therapeutic target of leptin signaling in breast cancer and cancer stem cells. Biochim Biophys Acta.

[5] Dubois V, Jardé T, Delort L, Billard H, Bernard-Gallon D, Berger E (2014). Leptin induces a proliferative response in breast cancer cells but not in normal breast cells. Nutr Cancer.

[6] Barone I, Catalano S, Gelsomino L, Marsico S, Giordano C, Panza S (2012). Leptin mediates tumor-stromal interactions that promote the invasive growth of breast cancer cells. Cancer Res.

[7] Vona-Davis L, Rose DP (2013). The obesity-inflammation-eicosanoid axis in breast cancer. J Mammary Gland Biol Neoplasia.

[8] Andò S, Catalano S (2012). The multifactorial role of leptin in driving the breast cancer microenvironment. Nat Rev Endocrinol.

[9] Chen X, Zha X, Chen W, Zhu T, Qiu J, Røe OD (2013). Leptin attenuates the anti-estrogen effect of tamoxifen in breast cancer. Biomed Pharmacother Bioméd Pharmacothérapie.

[10] Macciò A, Madeddu C (2011). Obesity, inflammation, and postmenopausal breast cancer: therapeutic implications. ScientificWorldJournal.

[11] Martínez-Martínez E, Jurado-López R, Valero-Muñoz M, Bartolomé MV, Ballesteros S, Luaces M (2014). Leptin induces cardiac fibrosis through galectin-3, mTOR and oxidative stress: potential role in obesity. J Hypertens.

[12] Valko M, Rhodes CJ, Moncol J, Izakovic M, Mazur M (2006). Free radicals, metals and antioxidants in oxidative stress-induced cancer. Chem Biol Interact.

[13] Adler V, Yin Z, Tew KD, Ronai Z (1999). Role of redox potential and reactive oxygen species in stress signaling. Oncogene.

[14] Sun Y, Huang L, Mackenzie GG, Rigas B (2011). Oxidative stress mediates through apoptosis the anticancer effect of phospho-nonsteroidal anti-inflammatory drugs: implications for the role of oxidative stress in the action of anticancer agents. J Pharmacol Exp Ther.

[15] Badid N, Ahmed FZB, Merzouk H, Belbraouet S, Mokhtari N, Merzouk SA (2010). Oxidant/antioxidant status, lipids and hormonal profile in overweight women with breast cancer. Pathol Oncol Res.

[16] Fernández-Sánchez A, Madrigal-Santillán E, Bautista M, Esquivel-Soto J, Morales-González A, Esquivel-Chirino C (2011). Inflammation, oxidative stress, and obesity. Int J Mol Sci.

[17] Nachat-Kappes R, Pinel A, Combe K, Lamas B, Farges M-C, Rossary A (2012). Effects of enriched environment on COX-2, Leptin and Eicosanoids in a Mouse Model of Breast Cancer. PloS One.

[18] Jardé T, Caldefie-Chézet F, Goncalves-Mendes N, Mishellany F, Buechler C, Penault-Llorca F (2009). Involvement of adiponectin and leptin in breast cancer: clinical and in vitro studies. Endocr Relat Cancer.

[19] Lamas B, Goncalves-Mendes N, Nachat-Kappes R, Rossary A, Caldefie-Chezet F, Vasson M-P (2013). Leptin modulates dose-dependently the metabolic and cytolytic activities of NK-92 cells. J Cell Physiol.

[20] Ru P, Steele R, Hsueh EC, Ray RB (2011). Anti-miR-203 upregulates SOCS3 expression in breast Cancer cells and enhances cisplatin Chemosensitivity. Genes Cancer.

[21] Butturini E, Darra E, Chiavegato G, Cellini B, Cozzolino F, Monti M (2014). S-Glutathionylation at Cys328 and Cys542 impairs STAT3 phosphorylation. ACS Chem Biol.

[22] Grossmann ME, Ray A, Nkhata KJ, Malakhov DA, Rogozina OP, Dogan S (2010). Obesity and breast cancer: status of leptin and adiponectin in pathological processes. Cancer Metastasis Rev.

[23] Loschen G, Azzi A, Richter C, Flohé L (1974). Superoxide radicals as precursors of mitochondrial hydrogen peroxide. FEBS Lett.

[24] Matés JM, Pérez-Gómez C, Núñez de Castro I (1999). Antioxidant enzymes and human diseases. Clin Biochemist.

[25] Hayes JD, Flanagan JU, Jowsey IR (2005). Glutathione transferases. Annu Rev Pharmacol Toxicol.

[26] Was H, Dulak J, Jozkowicz A (2010). Heme oxygenase-1 in tumor biology and therapy. Curr Drug Targets.

[27] Abraham NG, Kappas A (2008). Pharmacological and clinical aspects of Heme oxygenase. Pharmacol Rev.

[28] Balla J, Jacob HS, Balla G, Nath K, Eaton JW, Vercellotti GM (1993). Endothelial-cell heme uptake from heme proteins: induction of sensitization and desensitization to oxidant damage. Proc Natl Acad Sci.

[29] Lee W-Y, Chen Y-C, Shih C-M, Lin C-M, Cheng C-H, Chen K-C (2014). The induction of heme oxygenase-1 suppresses heat shock protein 90 and the proliferation of human breast cancer cells through its byproduct carbon monoxide. Toxicol Appl Pharmacol.

[30] Basu S. Bioactive eicosanoids: role of prostaglandin F (2α) and F_2_-isoprostanes in inflammation and oxidative stress related pathology. Mol Cells. 2010;30:383–91. 10.1007/s10059-010-0157-1 21113821

[31] Basu S, Nachat-Kappes R, Caldefie-Chézet F, Vasson M-P. Eicosanoids and adipokines in breast cancer: from molecular mechanisms to clinical considerations. Antioxid Redox Signal. 2013;18:323–60. 10.1089/ars.2011.4408 22746381

[32] Ghezzi P (2005). Oxidoreduction of protein thiols in redox regulation. Biochem Soc Trans.

[33] Lumb RA, Bulleid NJ (2002). Is protein disulfide isomerase a redox-dependent molecular chaperone?. EMBO J.

[34] Kargi A, Uysal M, Bozcuk H, Coskun HS, Savas B, Ozdogan M (2013). The importance of COX-2 expression as prognostic factor in early breast cancer. J BUON..

[35] Basu S. Bioactive eicosanoids: role of prostaglandin F(2α) and F_2_-isoprostanes in inflammation and oxidative stress related pathology. Mol. Cells. 2010;30:383–91. 10.1007/s10059-010-0157-1 21113821

[36] Shih R-H, Yang C-M (2010). Induction of heme oxygenase-1 attenuates lipopolysaccharide-induced cyclooxygenase-2 expression in mouse brain endothelial cells. J Neuroinflammation.

[37] Arab K, Rossary A, Flourié F, Tourneur Y, Steghens J-P (2006). Docosahexaenoic acid enhances the antioxidant response of human fibroblasts by upregulating gamma-glutamyl-cysteinyl ligase and glutathione reductase. Br J Nutr.

[38] Cheng WH, Ho YS, Ross DA, Han Y, Combs GFJ, Lei XG (1997). Overexpression of cellular glutathione peroxidase does not affect expression of plasma glutathione peroxidase or phospholipid hydroperoxide glutathione peroxidase in mice offered diets adequate or deficient in selenium. J Nutr.

[39] Cereser C, Guichard J, Drai J, Bannier E, Garcia I, Boget S (2001). Quantitation of reduced and total glutathione at the femtomole level by high-performance liquid chromatography with fluorescence detection: application to red blood cells and cultured fibroblasts. J Chromatogr B Biomed Sci App.

[40] Himmelfarb J, McMonagle E, McMenamin E (2000). Plasma protein thiol oxidation and carbonyl formation in chronic renal failure. Kidney Int.

[41] Arab K, Steghens J-P (2004). Plasma lipid hydroperoxides measurement by an automated xylenol orange method. Anal Biochem.

[42] Nalabolu MR, Palasamudram K, Jamil K (2014). Adiponectin and leptin molecular actions and clinical significance in breast cancer. Int J Hematol Oncol Stem Cell Res.

[43] Blanquer-Rosselló MM, Santandreu FM, Oliver J, Roca P, Valle A (2015). Leptin modulates mitochondrial function, dynamics and biogenesis in MCF-7 cells. J Cell Biochem.

[44] Bouloumie A, Marumo T, Lafontan M, Busse R (1999). Leptin induces oxidative stress in human endothelial cells. FASEB J.

[45] Sezgin Alikanoglu A, Yildirim M, Suren D, Yildiz M, Kaya V, Donem Dilli U (2014). Expression of cyclooxygenase-2 and Bcl-2 in breast cancer and their relationship with triple-negative disease. J BUON.

[46] Mazhar D, Ang R, Waxman J (2006). COX inhibitors and breast cancer. Br J Cancer.

[47] Chacón RD, Costanzo MV (2010). Triple-negative breast cancer. Breast Cancer Res.

[48] Martinez-Outschoorn U, Sotgia F, Lisanti MP (2014). Tumor microenvironment and metabolic synergy in breast cancers: critical importance of mitochondrial fuels and function. Semin Oncol.

[49] Kim DH, Song NY, Kim EH, Na HK, Joe Y, Chung HT (2014). 15-deoxy-∆12,14-prostaglandin J_2_ induces p53 expression through Nrf2-mediated upregulation of heme oxygenase-1 in human breast cancer cells. Free Radic Res.

[50] Tobar N, Cáceres M, Santibáñez JF, Smith PC, Martínez J (2008). RAC1 activity and intracellular ROS modulate the migratory potential of MCF-7 cells through a NADPH oxidase and NFkappaB-dependent mechanism. Cancer Lett.

[51] Xia C, Meng Q, Liu L-Z, Rojanasakul Y, Wang X-R, Jiang B-H (2007). Reactive oxygen species regulate angiogenesis and tumor growth through vascular endothelial growth factor. Cancer Res.

[52] Acharya A, Das I, Chandhok D, Saha T (2010). Redox regulation in cancer: a double-edged sword with therapeutic potential. Oxidative Med Cell Longev.

[53] Jiang F, Zhang Y, Dusting GJ (2011). NADPH oxidase-mediated redox signaling: roles in cellular stress response, stress tolerance, and tissue repair. Pharmacol Rev.

[54] Schaefer KN, Geil WM, Sweredoski MJ, Moradian A, Hess S, Barton JK (2015). Oxidation of p53 through DNA charge transport involves a network of disulfides within the DNA-binding domain. Biochemistry (Mosc).

[55] Wang D, Chen J, Chen H, Duan Z, Xu Q, Wei M (2012). Leptin regulates proliferation and apoptosis of colorectal carcinoma through PI3K/Akt/mTOR signalling pathway. J Biosci.

[56] Li N, Karin M (1999). Is NF-kappaB the sensor of oxidative stress?. FASEB J.

[57] Dirican N, Dirican A, Sen O, Aynali A, Atalay S, Bircan HA (2016). Thiol/disulfide homeostasis: a prognostic biomarker for patients with advanced non-small cell lung cancer?. Redox Rep Commun Free Radic Res.

[58] Leary PC O, Terrile M, Bajor M, Gaj P, Hennessy BT, Mills GB (2014). Peroxiredoxin-1 protects estrogen receptor alpha from oxidative stress-induced suppression and is a protein biomarker of favorable prognosis in breast cancer. Breast Cancer Res.

[59] Schafer FQ, Buettner GR (2001). Redox environment of the cell as viewed through the redox state of the glutathione disulfide/glutathione couple. Free Radic Biol Med.

[60] Esposito K, Ciotola M, Schisano B, Misso L, Giannetti G, Ceriello A (2006). Oxidative stress in the metabolic syndrome. J Endocrinol Investig.

[61] Tehan L, Taparra K, Phelan S (2013). Peroxiredoxin overexpression in MCF-7 breast cancer cells and regulation by cell proliferation and oxidative stress. Cancer Investig.

[62] Ray A, Nkhata KJ, Cleary MP (2007). Effects of leptin on human breast cancer cell lines in relationship to estrogen receptor and HER2 status. Int J Oncol.

[63] Maloberti PM, Duarte AB, Orlando UD, Pasqualini ME, Solano AR, López-Otín C (2010). Functional interaction between acyl-CoA synthetase 4, lipooxygenases and cyclooxygenase-2 in the aggressive phenotype of breast cancer cells. PLoS One.

